# Kinetics of *Leptospira interrogans* Infection in Hamsters after Intradermal and Subcutaneous Challenge

**DOI:** 10.1371/journal.pntd.0003307

**Published:** 2014-11-20

**Authors:** Mariana L. Coutinho, James Matsunaga, Long-Chieh Wang, Alejandro de la Peña Moctezuma, Michael S. Lewis, Jane T. Babbitt, Jose Antonio G. Aleixo, David A. Haake

**Affiliations:** 1 Veterans Affairs Greater Los Angeles Healthcare System, Los Angeles, California, United States of America; 2 Centro de Desenvolvimento Tecnologico, Universidade Federal de Pelotas, Pelotas, Brasil; 3 Faculdade de Veterinária, Universidade Federal do Rio Grande do Sul, Porto Alegre, Brasil; 4 Department of Medicine, David Geffen School of Medicine at UCLA, Los Angeles, California, United States of America; 5 CEIEPAA Facultad de Medicina Veterinaria y Zootecnia, Universidad Nacional Autónoma de México, Tequisquiapan, Querétaro, México; 6 Department of Urology, David Geffen School of Medicine at UCLA, Los Angeles, California, United States of America; 7 Departments of Microbiology, Immunology and Molecular Genetics, UCLA, Los Angeles, California, United States of America; Medical College of Wisconsin, United States of America

## Abstract

**Background:**

Leptospirosis is a zoonosis caused by highly motile, helically shaped bacteria that penetrate the skin and mucous membranes through lesions or abrasions, and rapidly disseminate throughout the body. Although the intraperitoneal route of infection is widely used to experimentally inoculate hamsters, this challenge route does not represent a natural route of infection.

**Methodology/Principal Findings:**

Here we describe the kinetics of disease and infection in hamster model of leptospirosis after subcutaneous and intradermal inoculation of *Leptospira interrogans* serovar Copenhageni, strain Fiocruz L1-130. Histopathologic changes in and around the kidney, including glomerular and tubular damage and interstitial inflammatory changes, began on day 5, and preceded deterioration in renal function as measured by serum creatinine. Weight loss, hemoconcentration, increased absolute neutrophil counts (ANC) in the blood and hepatic dysfunction were first noted on day 6. Vascular endothelial growth factor, a serum marker of sepsis severity, became elevated during the later stages of infection. The burden of infection, as measured by quantitative PCR, was highest in the kidney and peaked on day 5 after intradermal challenge and on day 6 after subcutaneous challenge. Compared to subcutaneous challenge, intradermal challenge resulted in a lower burden of infection in both the kidney and liver on day 6, lower ANC and less weight loss on day 7.

**Conclusions/Significance:**

The intradermal and subcutaneous challenge routes result in significant differences in the kinetics of dissemination and disease after challenge with *L. interrogans* serovar Copenhageni strain Fiocruz L1-130 at an experimental dose of 2×10^6^ leptospires. These results provide new information regarding infection kinetics in the hamster model of leptospirosis.

## Author Summary

Leptospirosis is the most widespread bacterial infection transmitted from animals to man. Humans are exposed to infection when host animals that harbor the bacteria in their kidneys shed them in their urine. Human infections, caused by the bacterium *Leptospira interrogans*, frequently result in a life-threatening illness characterized by liver and kidney failure. In the hamster model of leptospirosis, signs of hepatic and renal dysfunction developed on days 6 and 7, respectively, after intradermal and subcutaneous inoculation of *L. interrogans*. Renal dysfunction was preceded by the development of inflammatory changes and the appearance of large numbers of leptospires in the kidney on day 5. On day 6, animals began to lose weight, became dehydrated, and had elevated numbers of neutrophils circulating in their bloodstream. Importantly, animals inoculated intradermally had lower numbers of bacteria in their liver and kidneys on day 6 than animals inoculated subcutaneously and lower weight loss and circulating neutrophil levels on day 7. These studies show that the hamster model of leptospirosis is similar to human infection and indicate that the route of infection has significant effects on the course of the illness.

## Introduction

Leptospirosis is a zoonosis with worldwide distribution caused by spirochetes that are spread by reservoir animals to humans and other accidental hosts. Up to 500,000 severe human infections are documented annually in tropical areas with an incidence of >10 cases per 100,000 population [1]–[3]. Clinical severity ranges from asymptomatic to life-threatening disease characterized by hepatorenal failure with or without pulmonary hemorrhage. The mortality rate for severe leptospirosis ranges from 5–40% [4], [5]. Leptospirosis typically occurs in persons living in socioeconomically deprived conditions after exposure to flooding in humid, subtropical regions where conditions are favorable for environmental survival of the bacteria [6]–[8]. Contamination of water or soil occurs through urinary shedding by reservoir host animals. Although rodents are frequently the source of organisms causing human infections, many mammalian species have been found to harbor infection in their kidneys [9].

Acquisition of infection by a new host can occur through several possible routes, with cutaneous lesions as a major portal of entry [10]. The morphology and motility of leptospires and other spirochetes enhances their ability to penetrate across the skin and other tissue barriers, enter the bloodstream and disseminate to various organs via the endothelium [11]–[13]. Leptospirosis occurs as a dual-phase disease with a leptospiremic phase in the first week characterized by fever, myalgias, and other flu-like symptoms [14]. The immune phase begins as antibodies are produced and organisms are cleared from the bloodstream, associated with aseptic meningitis and recurrence of fever. In severe leptospirosis, the two phases may be obscured by the rapid progression of illness from its onset, leading to liver and kidney dysfunction characterized by jaundice and azotemia, respectively [4], [15]. Animal models have been shown to recapitulate the two stages of disease by examining the time to appearance of organisms in various tissues depending on the challenge route [16]–[19]. These initial studies primarily involved microscopic observations and isolation of leptospires from experimentally infected guinea pigs and hamsters, which are highly susceptible to infection with virulent leptospires. More recently, sensitive and quantitative PCR methods have been used to measure the leptospiral burden in various organs after intraperitoneal (IP) challenge [20], [21].

IP inoculation is the most common method employed in animal models of leptospirosis including hamsters and guinea pigs. Although this challenge route has certain technical advantages, it is not a natural route of infection as it bypasses relevant mucosal and cutaneous defense mechanisms. Other routes of inoculation such as epicutaneous, conjunctival, subcutaneous, intradermal, oral, intracardiac and intracranial have been reported [16], [20], [22]–[24], but quantification of leptospiral organ burden has only been described for the intraperitoneal route [20], [21]. Development of animal models that reproduce natural routes of transmission is critical to understanding host-pathogen interactions occurring early in leptospiral infection. In this study, we compared the kinetics of leptospiral infection after inoculation of hamsters via subcutaneous vs. intradermal routes of infection and compared changes in leptospiral burden in target organs with progression of disease as measured by body weight, changes in blood chemistry and vascular endothelial growth factor levels, histopathology, and antibody response.

## Materials and Methods

### Leptospiral strain and cultivation

*Leptospira interrogans* serovar Copenhageni strain Fiocruz L1-130 was cultivated in Ellinghausen-McCullough-Johnson-Harris (EMJH) medium [25], supplemented with 1% rabbit serum (Rockland Immunochemicals, Gilbertsville, PA) and 100 µg/ml 5-fluorouracil at 30°C in a shaker incubator (150 rpm). Leptospiral cultures (passage 2) in log phase of growth were centrifuged at 2,000×g for 5 minutes and resuspended in fresh EMJH prior to hamster inoculation.

### Hamster infection and sample collection

Female Syrian hamsters, 5 to 6 weeks of age (Harlan Bioscience, Indianapolis, IN), were inoculated either subcutaneously (0.5 mL) or intradermally (0.05 mL) using a 25 gauge needle with 2×10^6^ leptospires or EMJH alone on day 0. For intradermal inoculation, fur was removed with a pair of electric clippers to better visualize the injection site (Figure S1). From 3 to 4 animals were selected randomly for euthanasia from 1–9 days post-infection unless they presented with clinical signs of leptospirosis, such as loss of appetite, gait difficulty, dyspnea, prostration, ruffled fur, or weight loss of ≥10% of the animal's maximum weight. Kidney and liver were collected in formalin for histopathology or incubated overnight at 4°C in RNAlater (Ambion, Austin, TX), then RNAlater was decanted and tissue samples stored at −80°C. Paraffin embedded tissues were sectioned and stained with hematoxylin and eosin (H&E) or periodic acid-Schiff (PAS) in a Dako automated slide processor. Kidney sections were scored on a scale of 0 (normal tissue) to 5 (severe renal histopathology), based on the severity of glomerular injury, tubular cell damage, intratubular cast formation, interstitial inflammation, and capsular depression (up to 1 point for each). Blood was collected for serology, cell count, and chemical analyses (Antech Diagnostics, Irvine, CA). Vascular endothelial growth factor levels were measured using the Rodent MAP version 2.0 of Rules Based Medicine (Austin, Texas). All animal procedures were approved by the Veterans Affairs Greater Los Angeles Healthcare System, Institutional Animal Care and Use Committee (IACUC #04044-02) and adhered to the United States Health Research Extension Act of 1985 (Public Law 99–158, November 20, 1985, “*Animals in Research*”), the National Institutes of Health's *Plan for Use of Animals in Research* (Public Law 103-43, June 10, 1993), U.S. Government Principles for the Utilization and Care of Vertebrate Animals Used in Testing, Research, and Training, Public Health Service Policy on Humane Care and Use of Laboratory Animals, the United States Department of Agriculture's Animal Welfare Act & Regulations, and Veterans Health Administration Handbook 1200.7.

### DNA extraction and Quantitative PCR (qPCR)

Tissue DNA was extracted using either the FastDNA SPIN Kit (MP Biomedicals, Santa Ana, CA), accordingly to the manufacturer instructions or with the DNeasy Blood and Tissue kit (Qiagen, Valencia, CA), with modifications as previously described [26]. DNA quality was confirmed by measuring absorbance ratios at 260 nm vs 280 nm and also at 260 nm vs 230 nm. The purified DNA was stored at −80°C until use. DNA was tested by qPCR using the Bio-Rad iQ5 Real-time System (Bio-Rad, Hercules, CA). One hundred nanograms of total DNA were combined with 1 µM of each primer and 12.5 µL iQ SYBR Green Supermix (Bio-Rad) and brought to a final volume of 25 µL with nuclease-free water (Ambion, Austin, TX). Each sample was run in triplicate. qPCR primer pairs were LipL32-f, 5′-CGCTTGTGGTGCTTTCGGTG-3′, and LipL32-r, 5′- GCGCTTGTCCTGGCTTTACG-3′. The resulting amplicon was 152 bp. The PCR protocol consisted of an initial incubation step at 95°C for 12.5 min followed by 40 cycles of amplification (95°C for 15 s, 62°C for 30 s and 72°C for 30 s). Standard curves were generated ranging from 10 up to 1.6×106 copies of *Leptospira* (20-fold dilutions). DNA (100 ng) from 4 uninfected hamsters were used as negative controls.

### ELISA

96-well ELISA microtiter plates (Immulon 4HBX, Thermo Fisher, Waltham, MA) were coated with 1×10^9^ heat-inactivated leptospires/mL diluted in PBS, pH 7.2 (Invitrogen, Carlsbad, CA), by overnight incubation at 4°C as described previously [27]. Briefly, the plates were blocked with Protein-Free Blocking Buffer (PFBB, Thermo Fisher, Rockford, IL) for 1 to 2 h at room temperature (RT). Sera were tested in triplicate after 1∶6400 dilution with PFBB, to wells in a volume of 100 µL, and plates were incubated for 1 h at 37°C. After three washes in PFBB, wells were incubated with a 1∶5,000 dilution of peroxidase-conjugated anti-Syrian hamster IgG secondary antibody (Cat. #307-036-003, Jackson ImmunoResearch, West Grove, PA) for 30 min at RT. 100 µL of 1-Step Turbo Ultra TMB HRP substrate (Thermo Fisher) were added to the wells and incubated for 30 min at RT with shaking. The reaction was stopped by the addition of 50 µL of 2 M H_2_SO_4_ and plates were immediately read at 450 nm in a Bio-Rad 550 Microplate Reader.

### Statistics

One-way analysis of variance (ANOVA) with post-hoc Tukey and Šídák tests was used to test for differences between multiple (≥3) groups.

## Results

### Clinical response to infection and effect on body weight

**Figure 1:**
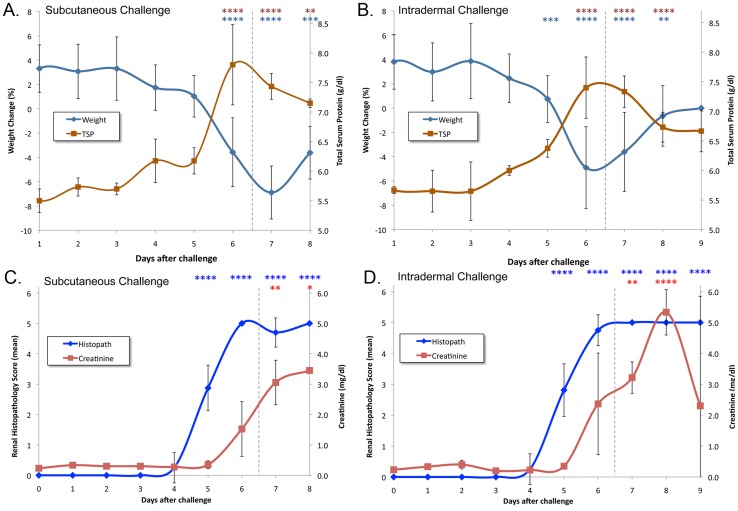
Kinetics of weight and kidney histopathology and function during leptospirosis infection. Animals were weighed at the time of subcutaneous (panels A and C) and intradermal (panels B and D) challenge (day zero) and daily thereafter. Panels A and B show mean change in weight relative to the previous day compared with the mean total serum protein (TSP) concentration showing weight loss and hemoconcentration beginning on day 6. Panels C and D show mean renal histopathology scores and mean serum creatinine, showing that abnormal histopathology preceded changes in kidney function tests. Error bars show standard deviation of the mean. Vertical dashed line indicates the point at which animals began to meet endpoint criteria. The number of asterisks indicate the level of significance compared to the control group (*P≤0.05, ** P≤0.01, ***P≤0.001, ****P≤0.0001). There were no differences between hamsters challenged subcutaneously and intradermally except for % weight change on day 7 after challenge.

We compared uninfected hamsters with animals challenged with 2×10^6^ leptospires via either the subcutaneous (SQ) or intradermal (ID) routes. Hamster body weight was included in the daily clinical examination. Uninfected hamsters gained an average of 3.6% of body weight per day during the course of the study. As shown in Figure 1, infected hamsters continued to gain weight until day 6 after challenge. A decrease in body weight was the earliest clinical sign of leptospirosis and, as in our previous study [26], the endpoint criterion of ≥10% weight loss prevented occurrence of spontaneous death. By day 7 after SQ challenge, 10/13 (77%) of the remaining hamsters had lost ≥10% of peak weight, and no hamsters survived to day 9 after challenge. By comparison, weight loss among ID challenged hamsters was not as severe; by day 7 after challenge, only 6/13 (46%) remaining animals had lost ≥10% of peak weight. The difference in weight loss on day 7 between the hamsters challenged ID vs. SQ was significant (P<0.01).

### Blood analysis

**Figure 2:**
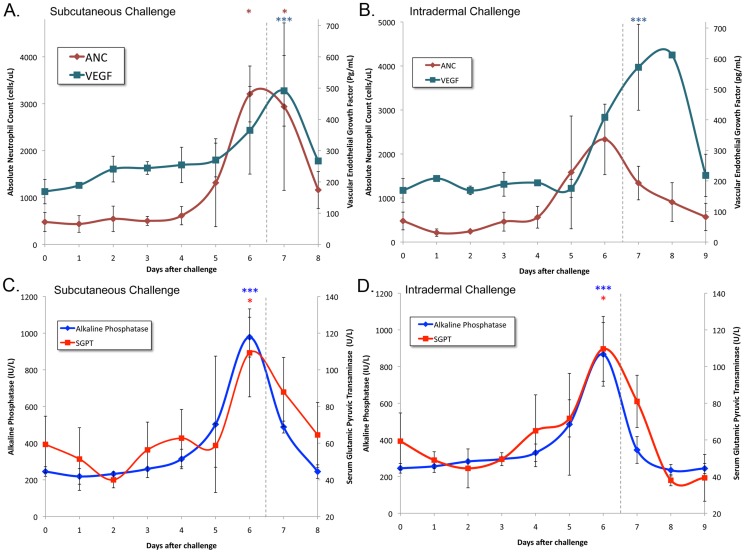
Kinetics of inflammatory markers and liver enzymes. Blood samples were collected at the time of subcutaneous (panels A and C) and intradermal (panels B and D) challenge (day zero) and daily thereafter. Panels A and B show mean absolute neutrophil counts (ANC) and vascular endothelial growth factor (VEGF) showing that the elevation in ANC on day 5 preceded the elevation in VEGF on day 6. Panels C and D show that the serum alkaline phosphatase and glutamic pyruvate transaminase (SGPT) peaked on day 6. Error bars show standard deviation of the mean. Vertical dashed line indicates the point at which animals began to meet endpoint criteria. The number of asterisks indicate the level of significance compared to the control group (*P≤0.05, ** P≤0.01, ***P≤0.001, ****P≤0.0001). There were no differences between hamsters challenged subcutaneously and intradermally except for the ANC on day 7 after challenge.

At the same time that hamsters began losing weight, total serum protein became elevated (Figure 1). The observed hemoconcentration is consistent with impairment in renal concentrating ability and nonoliguric renal insufficiency typically seen in the early stages of kidney disease due to leptospirosis [28]. Additional indications of the onset of impaired renal function on day 6 was the elevation in the serum creatinine (Figure 1), blood urea nitrogen (BUN) (Table S1) and blood phosphate levels (Table S1). Following the temporal pattern of weight loss and renal dysfunction, the absolute neutrophil count (ANC) and liver chemistries became elevated and peaked on day 6. As shown in Figure 2, the absolute neutrophil count (ANC) peaked at 5–6 fold over normal levels. After peaking on day 6, the ANC declined more rapidly after intradermal challenge compared to subcutaneous challenge (P<0.05). We found that uninfected hamsters had vascular endothelial growth factor (VEGF) levels of 169±39 pg/mL, which is similar to normal levels previously reported in mice [29], [30]. VEGF levels became elevated on day 6 and peaked on days 7–8. Like the ANC, the liver enzymes alkaline phosphatase (AP) and serum glutamic pyruvic transaminase (SGPT, alanine transaminase) peaked on day 6 (Figure 2). The total bilirubin level increased more slowly and was more variable among animals than the liver enzyme elevations (Table S1).

### Leptospiral organ burden

**Figure 3:**
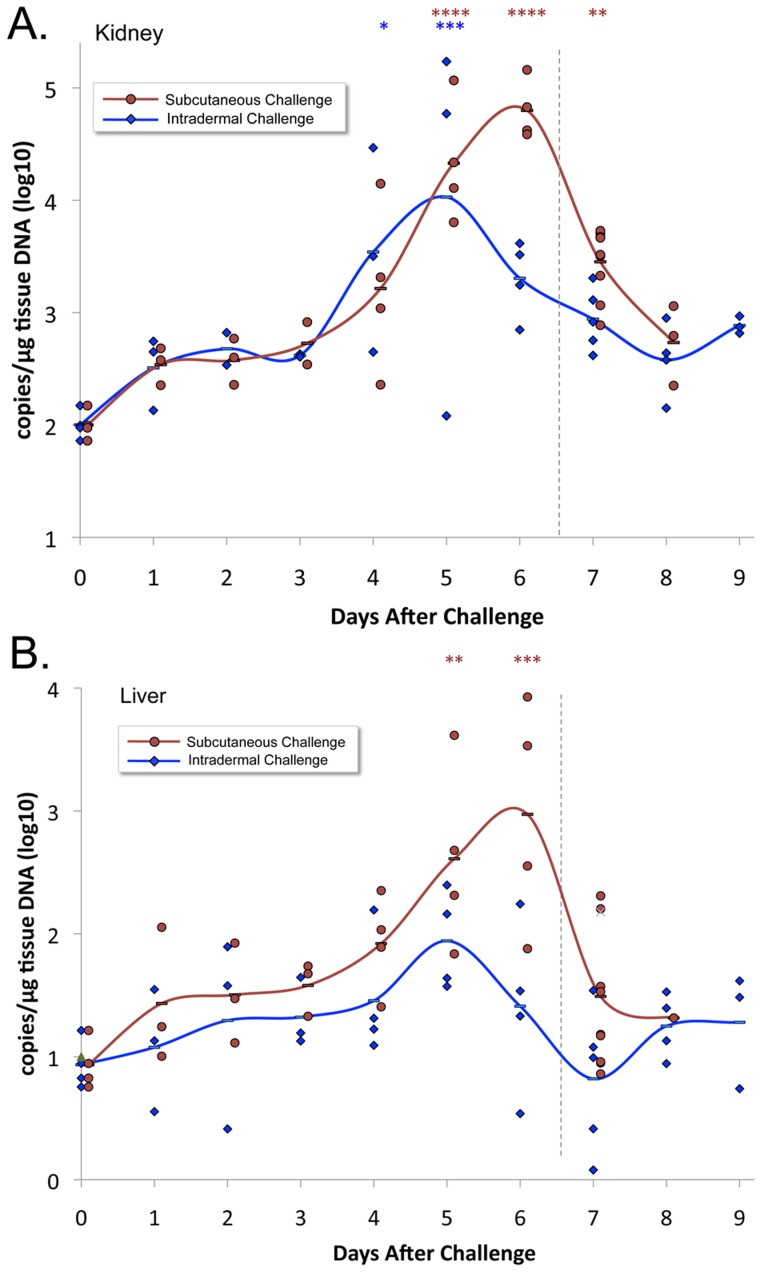
Leptospiral tissue burden. Kidney (panel A) and liver (panel B) tissues were subjected to DNA extraction and real-time PCR to measure the leptospiral copies per microgram of tissue DNA. Means (bars) and actual values (circles or diamonds) are depicted for animals challenged subcutaneously or intradermally. Vertical dashed line indicates the point at which animals began to meet endpoint criteria. The number of asterisks indicate the level of significance compared to the control group (*P≤0.05, ** P≤0.01, ***P≤0.001, ****P≤0.0001). Leptospiral tissue burdens after subcutaneous challenge were significantly higher than after intradermal challenge on day 6 in both the kidney and liver (P<0.01).

The leptospiral burden in kidneys and liver after SQ and ID challenge as measured by qPCR, in terms of DNA copies per microgram of tissue DNA, is presented in Figure 3. In animals infected by either route, the organ with the highest burden of organisms was the kidney. Significant numbers of organisms were found in the kidney as early as day 1 after SQ challenge and day 3 after ID challenge (t test, P<0.05). In animals challenged ID, the leptospiral burden in the kidney increased considerably after day 4 and reached a peak on day 5 of 8.5×10^3^ copies/µg of tissue DNA, after which the burden decreased. The leptospiral burden in the kidney after SQ challenge was similar to that after ID challenge through day 5. However, the leptospiral burden in the kidney continued to increase on day 6 after SQ challenge, peaking at a level six-fold higher (5.0×10^4^ copies/µg of tissue DNA), whereas it decreased on day 6 after ID challenge and was significantly lower than after SQ challenge (P<0.01). The leptospiral burden in the liver had a similar pattern to that in the kidney, reaching a peak on days 5 and 6 after ID and SQ challenge, respectively. However, in both challenge models, the peak leptospiral burden in the liver was two logs lower than it was in the kidney.

### Histopathology

**Figure 4:**
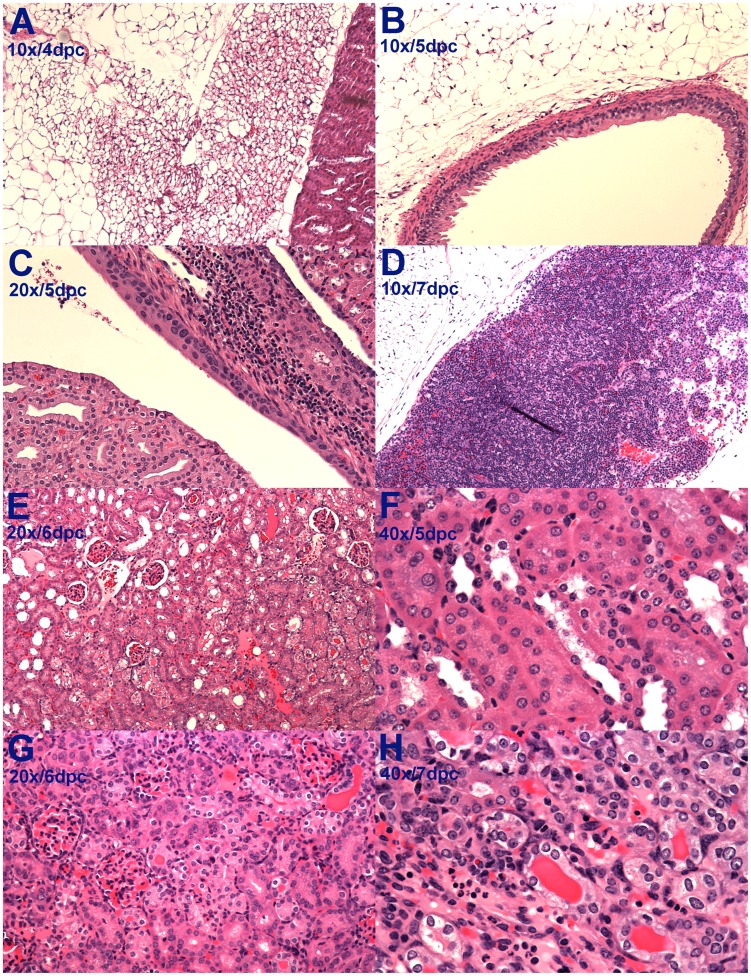
Histopathology changes in the kidney and perinephric tissues. A. Inflammatory changes in perinephric fat. B. Inflammatory changes in periureteral fat. C. Infiltration of inflammatory cells beneath the epithelium of the renal pelvis. D. Massively enlarged perihilar lymph node. E. Early glomerular and tubular damage with intratubular hyaline cast formation. F. Early interstitial inflammation in the kidney. G. Diffuse glomerular and tubular damage with hyaline cast formation. H. Severe tubular damage.

The histopathology findings were similar in the animals inoculated subcutaneously and intradermally. Kidney histology appeared normal until day 4 after challenge when mild focal tubular damage was noted in some animals. This early renal inflammation was mirrored by inflammatory changes in the perinephric (Fig. 4A) and periureteral (Fig. 4B) fat and in perihilar lymph nodes (Fig. 4D). On day 5, subpelvic inflammation was noted between the renal cortex and the transitional epithelium (Fig. 4C). By day 5, kidneys of all animals showed tubular damage with hyaline casts (Fig. 4E), interstitial infiltration of lymphocytes (Fig 4F) and glomerular damage with collapse of the Bowman's capsule (Fig. 4G). By day 6 and afterwards, these renal changes became both severe and diffuse (Fig. 4H) resulting in areas of capsular depression. The progression of disease was reflected by the increase in the histopathology score on day 5 (Fig. 1), which presaged the onset of renal dysfunction and weight loss by one day. Although occasional areas of hemorrhage were observed in the gross appearance of the liver, histologic changes were not observed.

### Antibody response

**Figure 5:**
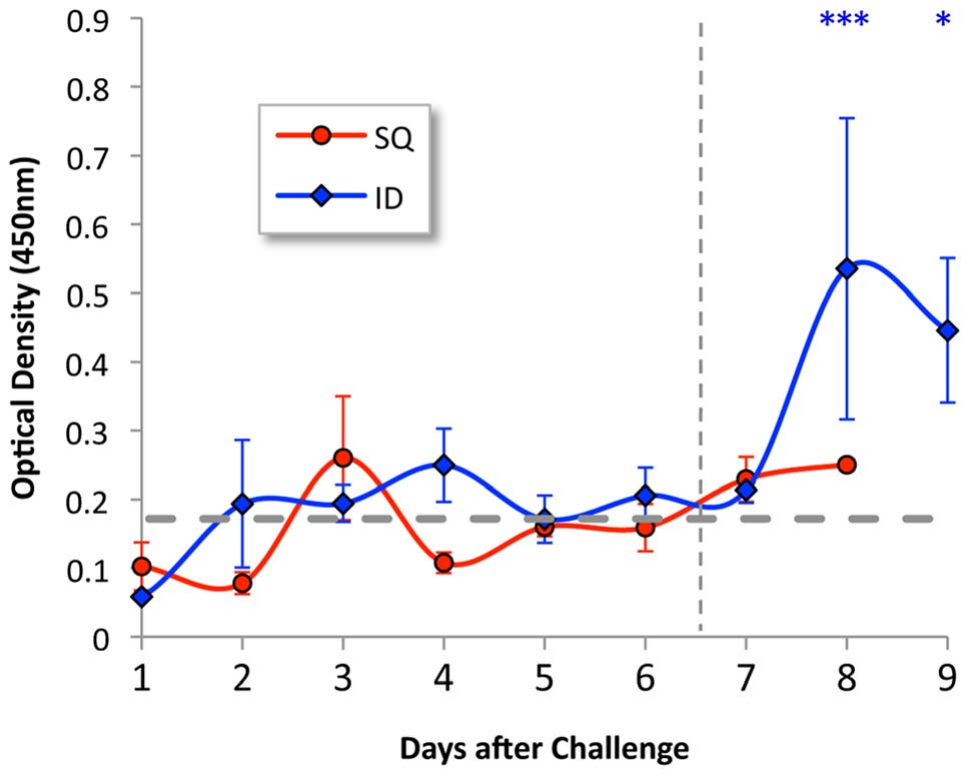
Antibody response in infected hamsters. Hamster immunoglobulin responses to heat-killed leptospires were measured by ELISA. Mean and standard errors are shown for serum samples (tested in triplicate at a dilution of 1∶6400) obtained from hamsters challenged intradermally (red) or subcutaneously (blue). Dotted gray line represents two standard deviations above background ELISA for sera from six uninfected control hamsters. Vertical dashed line indicates the point at which animals began to meet endpoint criteria. The number of asterisks indicate the level of significance compared to the control group (*P≤0.05, ***P≤0.001). Leptospiral antibody levels after intradermal challenge were significantly higher than after subcutaneous challenge on day 8 (P<0.05).

Animals challenged by the SQ route did not show an increase in antibody level during the 8 days of the experiment. As shown in Figure 5, animals challenged by the ID route were found to form significant antibody levels on days 8 and 9 after challenge.

## Discussion

The hamster is one of most widely used animal models of acute leptospirosis infection because of its reproducibility and the susceptibility of hamsters to a wide variety of pathogenic *Leptospira* strains [16], [19], [31], [32]. However, little is known about the relative burden of organisms in key organs such as the kidney and liver, and the role of leptospiral burden in disease [20]. For this reason, we were interested in examining the time course of infection relative to an array of disease parameters including body weight, histopathology, inflammatory markers, and markers of renal and hepatic function. Organisms were detected in the kidney by qPCR at levels significantly above background by days 1 and 3 after infection by the SQ and ID routes, respectively. Beginning on day 4 or 5 after infection by either the ID or SQ routes, we observed a major increase in leptospiral burden in the kidney, with the appearance of renal and perinephric histopathology changes on day 5. These histopathology changes were followed one day later (day 6) by deterioration in kidney function, as demonstrated by an elevation in serum creatinine, and the onset of weight loss. The rapid progression from infection to disease that we observed in hamsters mimics the lack of a biphasic pattern of illness seen in severe human leptospirosis. Daily measurement of hamster weight provides a sensitive and objective measure of health, and as in our previous study [26], utilization of ≥10% weight loss from maximum weight as an endpoint criterion completely prevented spontaneous death. Weight loss occurred simultaneously with elevation of total serum proteins, indicating that these changes were due to leptospirosis-induced dehydration and hemoconcentration through decreased fluid intake and impairment of sodium reabsorption in the renal proximal tubules [28].

The peak in the burden of infection on days 5 and 6, followed closely by or concurrent with the onset of weight loss, elevation of inflammatory markers, and changes in blood analytical chemistry results indicative of renal and hepatic dysfunction, implies a causal relationship. Elevation in the absolute neutrophil count (ANC) is a typical response to systemic infection that is also observed in human leptospirosis [4]. In our study, the ANC was found to peak at 5- to 6-fold over the normal values for uninfected hamsters (Figure 2). It is unclear what role neutrophils had in the subsequent decrease in the burden of organisms in the kidney and liver. In vitro studies suggest that neutrophils are unable to kill pathogenic leptospires unless specific antibody is present [33], [34]. However, we were not able to detect circulating leptospiral antibodies until day 8 after infection, and only in animals challenged intradermally (Figure 5). It is possible that leptospiral antibodies were not detectable in the serum at earlier timepoints because antibodies were complexed with organisms due to a state of antigen excess. The association between detectable anti-leptospiral antibody levels and lower tissue burdens of organisms in intradermally challenged hamsters is consistent with a known protective role for the humoral immune response in leptospirosis [35].

Levels of vascular endothelial growth factor (VEGF) were examined as an additional marker of inflammation. Elevated VEGF levels, as documented in our study, have been found to be an important determinant of morbidity and mortality in the mouse model of sepsis [36]. Kidney and liver dysfunction are hallmarks of the severe form of leptospirosis known as Weil's syndrome. As shown in Figures 1 and 2, deterioration in renal function and elevation in liver enzymes mirrored the peak in leptospiral burden in the kidneys and liver. We have previously shown high numbers of leptospires in the glomeruli and hepatic sinusoids during acute leptospirosis [37] and others have shown that leptospiral infection leads to hepatocyte apoptosis as a mechanism of liver dysfunction in the guinea pig model of leptospirosis [38].

**Figure 6:**
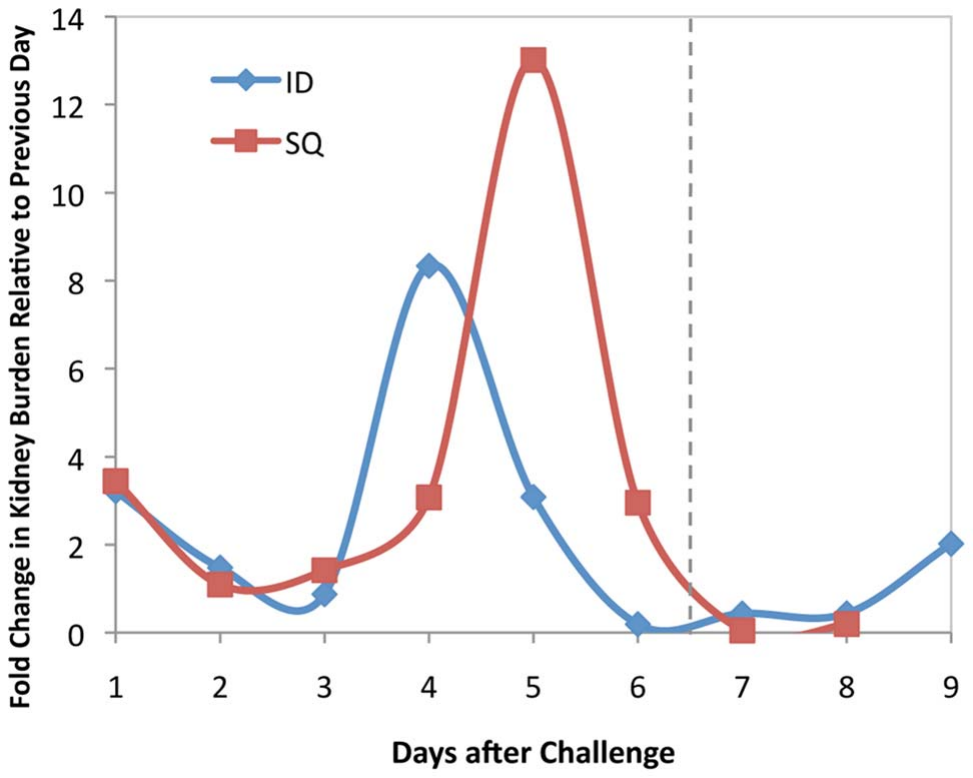
Fold change in kidney burden. The fold change in kidney burden relative to the previous day as determined by qPCR was plotted for animals challenged intradermally (ID) vs. subcutaneously (SQ). There was a one-day surge in the burden of organisms in the kidney several days after challenge that was larger and later in animals challenged via the SQ route as compared to the ID route.

The qPCR approach allowed us to document striking 8- and 13-fold increases in leptospiral burden in the kidney between days 3 and 4 after ID challenge and between days 4 and 5 after SQ challenge, respectively. As shown in Figure 6, these dramatic increases in burden of infection in the kidney are in stark contrast to relatively low rates of change on other days suggesting delivery of organisms to the kidney from the bloodstream and/or multiplication within the kidney. A similar increase in the burden of infection in the kidney between days 5 and 6 after ip challenge has also been reported by Lourdault *et al*. [20], although in that guinea pig study, qPCR levels were significantly higher in the liver than the kidney. In contrast, we observed a higher burden of organisms in the kidney than the liver. These differences in organ predilection could be due to a variety of factors including the virulence of the leptospiral strain, the host animal species, the route of infection and/or immune-mediated delivery mechanisms.

A limitation of this study is that leptospiremia levels were not measured in the blood. However, bacterial burdens were measured in the spleen, and given its role in blood filtration, the spleen can be considered as a surrogate for the blood. Another limitation is that some of the bacterial burden found in organs was due to residual blood, given that animals were not perfused with saline prior to organ harvesting. Nevertheless, bacterial burdens were higher in the kidney than in either the spleen or liver by more than an order of magnitude. These data indicate that leptospires selectively target the kidneys during infection and substantiate the contribution of the higher bacterial burdens to the pathogenesis of kidney dysfunction. The predilection for kidney infection was observed at early time points. Organisms were detected in the kidney by qPCR at levels significantly above background by day 1 and 3 after infection by the SQ and ID routes, respectively. Homing to the kidney by leptospires may be mediated either by leptospiral factors such as kidney-specific adhesins or by production of anti-leptospiral antibodies. Colonization of the lumen of proximal renal tubules has been documented by immunohistochemistry as early as 4 days after intraperitoneal challenge [39].

A novel aspect of our study was the comparison of the SQ and ID challenge routes with the finding of significant differences in the kinetics of infection. The differences in leptospiral tissue burden were most pronounced on day 6, when we observed 31- and 36-fold higher levels of leptospiral DNA in kidney and liver tissues respectively, after SQ compared to ID inoculation. The lower leptospiral burden in kidney and liver was reflected in less weight loss on day 7 and better survival in ID challenged animals. The ID route is of interest because it is a biologically relevant route of infection in individuals with abrasions and cutaneous exposure. An ID challenge model incorporates interactions between leptospires and defense mechanisms of the skin, such as the dendritic cells, that may function as an early warning system for the immune response. Prompt immune activation during the earliest stages of the transmission process is to the advantage of the host if it results in early clearance of organisms and a lower leptospiral burden. The intradermal route may also be of interest as a more reliable way to evaluate transmission-blocking vaccines using surface antigens that are either expressed in the environmental phase or early in infection.

## Supporting Information

Figure S1 Intradermal inoculation of hamsters. Hamsters were anesthetized by isoflurane inhalation, shaved on the left flank of their abdomen then infected intradermally with 100 µl of leptospiral culture. ID injection induces a bleb at the site of inoculation.
(TIF)

Table S1 Data compilation of serum chemistry values, blood counts, and vascular endothelial growth factor levels for control animals and animals inoculated either subcutaneously (SQ) or intradermally (ID) on each day of the study.
(PDF)
