# Supplementary figures and images for: Kinetics of Leptospira interrogans Infection in Hamsters after Intradermal and Subcutaneous Challenge
Source: PLoS Negl Trop Dis. 2014 Nov 20;8(11):e3307. doi: 10.1371/journal.pntd.0003307 (PMC4239013; doi:10.1371/journal.pntd.0003307)

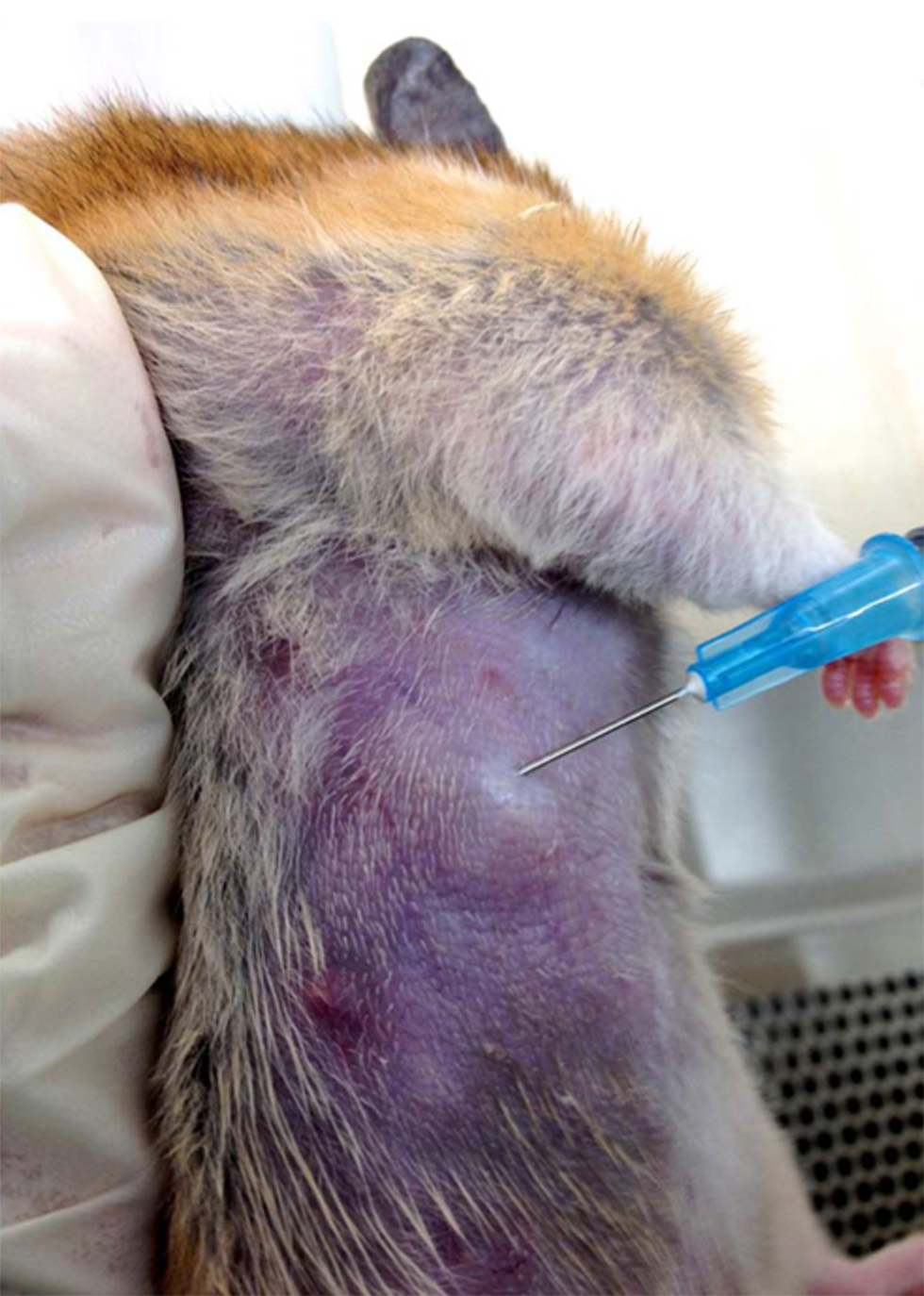

Supplement: Figure S1 — Intradermal inoculation of hamsters. Hamsters were anesthetized by isoflurane inhalation, shaved on the left flank of their abdomen then infected intradermally with 100 µl of leptospiral culture. ID injection induces a bleb at the site of inoculation. (TIF) [file pntd.0003307.s001.tif]
