# Supplementary material for: Kinetics of Leptospira interrogans Infection in Hamsters after Intradermal and Subcutaneous Challenge
Source: PLoS Negl Trop Dis. 2014 Nov 20;8(11):e3307. doi: 10.1371/journal.pntd.0003307 (PMC4239013; doi:10.1371/journal.pntd.0003307)
Supplement: Table S1 — Data compilation of serum chemistry values, blood counts, and vascular endothelial growth factor levels for control animals and animals inoculated either subcutaneously (SQ) or intradermally (ID) on each day of the study. (PDF) [file pntd.0003307.s002.pdf]

Table S1

	Route		C	1	2	3	4	5	6	7	8	9
			**Days after Challenge**									
Alkaline Phosphatase (IU/L)	SQ	mean	245.3	218.7	232.3	259.3	313.8	502.8	978.3	487.9	245.0	ND
		SD	26.0	42.3	11.7	3.8	53.3	371.9	108.5	32.5	36.8	ND
	ID	mean	245.3	256.3	282.5	295.5	330.0	485.3	867.0	345.3	235.0	244.3
		SD	26.0	33.5	3.5	0.7	47.7	277.6	173.7	73.1	31.4	27.5
Total Bilirubin (mg/dL)	SQ	mean	0.1	0.1	0.1	0.1	0.2	0.1	0.8	2.1	1.9	ND
		SD	0.0	0.0	0.0	0.0	0.1	0.0	0.4	2.8	2.3	ND
	ID	mean	0.1	0.1	0.1	0.2	0.1	0.1	0.7	0.3	0.3	0.2
		SD	0.0	0.0	0.0	0.1	0.1	0.0	0.7	0.2	0.1	0.1
SGPT (U/L)	SQ	mean	59.3	51.3	40.0	56.3	62.8	58.8	109.3	87.9	64.5	ND
		SD	15.4	17.1	4.4	15.1	15.6	11.9	24.0	18.9	17.7	ND
	ID	mean	59.3	49.0	44.5	49.5	65.0	71.8	109.7	81.0	38.0	39.3
		SD	15.4	4.6	10.6	3.5	19.6	10.1	17.7	14.3	3.0	12.7
Total Serum Protein (g/dl)	SQ	mean	6.0	5.5	5.7	5.7	6.2	6.2	7.8	7.4	7.2	ND
		SD	0.2	0.2	0.2	0.1	0.4	0.2	0.7	0.2	0.1	ND
	ID	mean	6.0	5.7	5.7	5.7	6.0	6.4	7.4	7.3	6.7	6.7
		SD	0.2	0.1	0.4	0.5	0.1	0.2	0.5	0.3	0.3	0.4
Calcium (mg/dL)	SQ	mean	15.2	13.9	13.4	13.4	13.9	13.4	13.9	12.1	11.5	ND
		SD	0.5	0.4	0.6	0.4	0.6	0.5	0.8	1.2	0.2	ND
	ID	mean	15.2	14.2	13.3	12.8	14.5	14.4	13.7	11.4	11.5	13.0
		SD	0.5	0.5	0.2	1.7	0.9	0.5	2.5	1.9	0.5	2.6
Phosphorus (mg/dL)	SQ	mean	11.9	23.8	22.8	22.0	24.1	21.8	44.8	28.1	22.2	ND
		SD	0.8	3.7	5.2	2.5	4.3	5.8	7.2	2.7	1.2	ND
	ID	mean	11.9	25.1	28.0	22.3	20.1	22.4	40.8	27.1	24.2	15.9
		SD	0.8	4.2	9.1	7.2	4.7	1.9	13.3	2.6	1.4	6.1
Glucose (mg/dL)	SQ	mean	75.7	14.7	11.7	11.3	11.8	17.3	10.0	10.0	10.0	ND
		SD	35.2	8.1	2.9	2.3	2.4	8.4	0.0	0.0	0.0	ND
	ID	mean	75.7	22.7	10.0	10.0	13.3	65.3	20.7	10.0	43.0	12.3
		SD	35.2	21.9	0.0	0.0	6.5	69.6	11.0	0.0	30.4	3.2
Creatinine (mg/dl)	SQ	mean	0.2	0.3	0.3	0.3	0.3	0.4	1.5	3.1	3.5	ND
		SD	0.1	0.1	0.1	0.0	0.1	0.1	0.9	0.7	0.1	ND
	ID	mean	0.2	0.3	0.4	0.2	0.2	0.4	2.4	3.2	5.3	2.3
		SD	0.1	0.1	0.1	0.0	0.0	0.1	1.6	0.5	0.7	3.6
Blood Urea Nitrogen (mg/dL)	SQ	mean	19.3	24.3	23.7	23.0	22.8	26.8	77.0	176.3	191.0	ND
		SD	2.3	2.1	2.5	3.5	1.0	8.5	38.4	55.7	48.1	ND
	ID	mean	19.3	24.0	23.0	21.5	22.0	26.8	107.7	226.2	322.7	129.0
		SD	2.3	2.6	0.0	3.5	3.6	3.0	71.8	79.8	57.7	169.8
White Blood Count (x1000/µL)	SQ	mean	2.9	3.3	2.8	3.5	3.1	4.5	6.0	8.2	4.0	ND
		SD	0.6	1.7	0.5	0.6	1.2	1.7	0.2	3.2	0.8	ND
	ID	mean	2.9	2.1	1.5	3.4	3.0	4.2	5.6	4.9	3.1	2.5
		SD	0.6	0.8	0.8	0.9	0.3	1.5	1.3	1.8	1.9	0.3
Neutrophils (%)	SQ	mean	16.0	14.7	15.7	17.3	20.3	27.8	54.0	32.9	29.0	ND
		SD	4.0	5.0	5.9	2.5	4.1	9.3	10.5	14.3	4.2	ND
	ID	mean	16.0	10.0	19.0	13.0	18.3	33.5	45.0	28.2	25.7	23.0
		SD	4.0	0.0	9.9	3.0	6.7	18.6	22.9	5.7	2.1	12.1
Lymphocytes (%)	SQ	mean	76.7	82.3	80.7	79.3	76.3	69.3	37.3	59.8	63.5	ND
		SD	8.1	5.0	6.4	2.5	4.2	9.3	12.1	16.0	6.4	ND
	ID	mean	76.7	65.0	49.0	63.8	63.2	51.2	35.8	56.7	53.0	56.3
		SD	8.1	1.2	17.7	3.0	6.6	19.3	25.7	4.4	1.7	11.5

Monocytes (%)	SQ	mean	5.0	2.0	2.7	2.3	2.8	2.0	7.3	6.3	6.5	ND
		SD	2.0	0.0	1.2	0.6	1.0	0.0	3.3	2.2	2.1	ND
	ID	mean	5.0	2.0	6.0	2.0	2.8	2.5	8.7	5.8	5.3	3.7
		SD	2.0	0.0	5.7	0.0	0.5	0.6	3.8	1.9	0.6	2.1
Eosinophils (%)	SQ	mean	2.3	1.0	1.0	1.0	1.0	1.0	1.5	1.0	1.0	ND
		SD	2.3	0.0	0.0	0.0	0.0	0.0	1.3	0.0	0.0	ND
	ID	mean	2.3	1.7	2.5	1.0	1.0	1.3	0.7	1.0	1.0	1.3
		SD	2.3	1.2	2.1	0.0	0.0	0.5	0.6	0.0	0.0	0.6
Red Blood Cells (xmillion/µL)	SQ	mean	7.8	5.9	6.9	7.0	6.6	7.5	8.4	8.7	8.1	ND
		SD	0.4	1.8	0.2	0.2	1.6	0.3	0.5	0.4	0.4	ND
	ID	mean	7.8	4.7	3.5	7.1	7.2	7.6	8.2	8.4	7.7	7.7
		SD	0.4	1.7	2.4	0.3	0.5	0.7	0.6	0.1	0.1	0.7
Hematocrit (%)	SQ	mean	54.0	40.7	47.0	49.0	44.3	48.0	55.0	57.8	53.5	ND
		SD	2.6	12.7	1.0	2.0	10.8	2.2	3.4	2.5	3.5	ND
	ID	mean	54.0	33.0	23.0	47.3	49.3	48.8	55.3	55.7	52.3	52.0
		SD	2.6	11.5	15.6	2.1	4.3	4.1	0.6	1.5	1.5	3.6
Hemoglobin (g/dL)	SQ	mean	16.0	12.5	14.6	15.0	13.8	15.4	17.4	17.5	16.4	ND
		SD	0.9	3.9	0.2	0.5	3.3	0.6	1.0	0.8	1.2	ND
	ID	mean	16.0	10.0	7.3	14.9	15.1	15.6	17.6	16.8	15.7	15.8
		SD	0.9	3.7	5.2	0.6	1.1	1.6	0.6	0.5	0.1	1.1
MCH (pg/cell)	SQ	mean	20.5	21.1	21.1	21.3	21.0	20.8	20.7	20.2	20.3	ND
		SD	0.6	0.2	0.3	0.2	0.3	0.5	0.5	0.4	0.5	ND
	ID	mean	20.5	21.0	20.8	21.0	20.9	20.6	21.4	20.1	20.3	20.6
		SD	0.6	0.5	0.4	0.2	0.2	0.5	1.3	0.5	0.2	0.3
MCHC (g/dL)	SQ	mean	29.7	30.7	31.3	31.0	31.3	32.3	31.8	30.6	31.0	ND
		SD	0.6	0.6	0.6	0.0	0.5	0.5	0.5	0.5	0.0	ND
	ID	mean	29.7	30.7	31.0	31.7	30.8	32.0	31.7	30.0	30.3	30.7
		SD	0.6	0.6	1.4	0.6	0.5	0.0	0.6	0.0	1.2	0.6
MCV (fL/red cell)	SQ	mean	69.0	68.7	68.0	69.3	67.3	64.8	65.8	66.6	66.0	ND
		SD	2.6	1.5	2.0	1.5	1.3	1.7	1.0	1.6	1.4	ND
	ID	mean	69.0	68.7	66.5	67.0	68.0	64.5	64.7	66.5	67.7	67.7
		SD	2.6	1.5	2.1	0.0	0.8	1.7	2.9	1.5	2.1	0.6
Platelets* (x1000/µL)	SQ	mean	1128	307	448	350	818	614	678	563	439	ND
		SD	155	112	222	41	441	142	270	274	41	ND
	ID	mean	1128	223	229	380	454	951	380	895	832	1255
		SD	155	119	170	84	73	484	69	382	398	475
VEGF (pg/mL)	SQ	mean	169	189	241	244	254	270	365	491	267	ND
		SD	39	17	4	15	16	12	24	19	18	ND
	ID	mean	169	208	169	189	194	176	408	572	612	218
		SD	39	0	14	39	10	30	0	141	0	69

*Reflects the minimum number due to platelet clumping.
